# P32. High resolution mass spectrometry reveals the depth and diversity of HLA-I peptidomes

**DOI:** 10.1186/2051-1426-2-S2-P23

**Published:** 2014-03-12

**Authors:** M Bassani-Sternberg, S Frankild, M Mann

**Affiliations:** 1 Max Planck Institute for Biochemistry, Proteomics and Signal Transduction, Martinsried/Munich, Germany; 2 Novo Nordisk Foundation Center for Protein Research (CPR), Faculty of Health, Copenhagen, Denmark

## 

T-cell responses against infected and cancer cells are initiated by recognition of HLA-I peptides (the peptidome) presented on the surface of nucleated cells. The repertoire of HLA-I peptides originates primarily from sampling the cytosolic degradation products of intracellular proteins. HLA-I peptides have been extensively studied in the last years because of their immediate use as immunotherapy based cancer vaccines. Even more advanced cell based therapeutic applications are being developed based on cancer specific HLA-I peptides. In this study, we used high resolution mass spectrometry and the MaxQuant bioinformatics environment to obtain a high accuracy and in-depth coverage of HLA peptidomes. HLA-I peptidomes were immuno-affinity purified from 10 cancer and primary cell lines. The unprecedented high number of thousands of identified HLA-I peptides per cell line enabled us to evaluate the known mechanisms governing peptidome presentation and to determine the proteome which is sampled for presentation. We envision that applying our methodology for analysing patients tumour samples will result in the discovery of new cancer specific peptides. Better immunotherapeutic modules could possibly be developed based on wider and more accurate repertoires of HLA-I peptides and they will increase the accessibility of these therapies for a larger cohort of patients.

